# Pharmacological treatment and BBB-targeted genetic therapy for MCT8-dependent hypomyelination in zebrafish

**DOI:** 10.1242/dmm.027227

**Published:** 2016-11-01

**Authors:** David Zada, Adi Tovin, Tali Lerer-Goldshtein, Lior Appelbaum

**Affiliations:** The Faculty of Life Sciences andthe Multidisciplinary Brain Research Center, Bar-Ilan University, Ramat-Gan 5290002, Israel

**Keywords:** Psychomotor-retardation, Live imaging, Zebrafish, Myelin, Thyroid, Mct8, Blood–brain barrier, Allan-Herndon-Dudley syndrome (AHDS)

## Abstract

Hypomyelination is a key symptom of Allan-Herndon-Dudley syndrome (AHDS), a psychomotor retardation associated with mutations in the thyroid-hormone (TH) transporter MCT8 (monocarboxylate transporter 8). AHDS is characterized by severe intellectual deficiency, neuromuscular impairment and brain hypothyroidism. In order to understand the mechanism for TH-dependent hypomyelination, we developed an *mct8* mutant (*mct8*^−/−^) zebrafish model. The quantification of genetic markers for oligodendrocyte progenitor cells (OPCs) and mature oligodendrocytes revealed reduced differentiation of OPCs into oligodendrocytes in *mct8*^−/−^ larvae and adults. Live imaging of single glial cells showed that the number of oligodendrocytes and the length of their extensions are reduced, and the number of peripheral Schwann cells is increased, in *mct8*^−/−^ larvae compared with wild type. Pharmacological analysis showed that TH analogs and clemastine partially rescued the hypomyelination in the CNS of *mct8*^−/−^ larvae. Intriguingly, triiodothyronine (T3) treatment rescued hypomyelination in *mct8*^−/−^ embryos before the maturation of the blood–brain barrier (BBB), but did not affect hypomyelination in older larvae. Thus, we expressed Mct8-tagRFP in the endothelial cells of the vascular system and showed that even relatively weak mosaic expression completely rescued hypomyelination in *mct8*^−/−^ larvae. These results suggest potential pharmacological treatments and BBB-targeted gene therapy that can enhance myelination in AHDS and possibly in other TH-dependent brain disorders.

## Abstract (precis)

**Summary:** Expression of thyroid transporter specifically in the blood–brain barrier of zebrafish, and pharmacological treatments with thyroid hormone analogs and clemastine, rescue myelin deficiencies.

## INTRODUCTION

Leukodystrophies are a group of genetic disorders that affect the central nervous system (CNS) by altering the development and maintenance of myelin. Hypomyelinating leukodystrophies are caused by a deficiency in myelin deposition and are characterized by developmental delay, hypotonia, spasticity and various intellectual disabilities (Boespflug-Tanguy et al., 2008; Charzewska et al., 2016). Myelination is a process in which specialized glial cells – oligodendrocytes in the CNS and Schwann cells in the peripheral nervous system (PNS) – send extensions of fatty substance and form myelin sheaths that wrap axons. This insulation is vital for rapid electrical conduction and information processing (Hartline and Colman, 2007; Raphael and Talbot, 2011; Czopka, 2016). The functional myelin-producing cells are differentiated from oligodendrocyte progenitor cells (OPCs), which are active primarily during embryonic development but also in juveniles and adults (Dawson et al., 2003). Although hypomyelination disorders are extensively studied, the pathogenic mechanism is unclear and treatments are limited.

Among hypomyelination leukodystrophies, the X-linked Allan-Herndon-Dudley syndrome (AHDS) is a psychomotor retardation characterized by severe intellectual deficiency, neuromuscular impairment and altered thyroid hormone (TH) levels in the serum (Friesema et al., 2004; Dumitrescu et al., 2004; Brockmann et al., 2005). Diagnosis using magnetic resonance imaging (MRI) showed a global lack of cerebral white matter in AHDS patients (Gika et al., 2010; Holden et al., 2005; La Piana et al., 2015). AHDS is associated with mutations in monocarboxylate transporter 8 (*MCT8*, *SLC16A2*), which transports TH across the cell membrane (Ceballos et al., 2009; Friesema et al., 2003). MCT8 is primarily expressed in the CNS, vascular system and blood–brain barrier (BBB) (Friesema et al., 2012; Pizzagalli et al., 2002; Roberts et al., 2008). In order to study the mechanism underlying AHDS, an *Mct8* knockout (*Mct8*-KO) mouse model was established. Similar to individuals with AHDS, the *Mct8*-KO mice showed altered TH levels in the serum; however, neurological and behavioral phenotypes were not apparent (Di Cosmo et al., 2013; Dumitrescu et al., 2006; Rodrigues et al., 2013; Trajkovic et al., 2007). This might be explained by a compensation mechanism in mice in which the organic anion transporting polypeptide 1C1 (*Oatp1c1*), a T4-selective transporter, is predominantly expressed in the BBB (Ito et al., 2011; Mayerl et al., 2012; Roberts et al., 2008). Indeed, *Mct8/Oatp1c1* double-KO (dKO) mice display both endocrinological and neurological phenotypes found in humans with AHDS, including hypomyelination (Mayerl et al., 2014). Nevertheless, it is unclear why a lack of MCT8 causes hypomyelination, and understanding the developmental mechanisms could provide the groundwork to develop genetic and pharmacological treatments for AHDS and, potentially, other hypomyelination leukodystrophies.

In order to study AHDS and hypomyelination, we used the zebrafish model, which combines invertebrate-like genetics with vertebrate brain structures (Elbaz et al., 2015; Levitas-Djerbi et al., 2015; Yelin-Bekerman et al., 2015), and its transparency allows real-time imaging of myelination in a live animal (Kirby et al., 2006; Buckley et al., 2008). In addition, zebrafish larvae have emerged as an attractive model for genetic manipulations and high-throughput therapeutic drug screens (Kaufman et al., 2009; MacRae and Peterson, 2015; Tsuji et al., 2014). The zebrafish *mct8* gene and promoter were isolated (Arjona et al., 2011; Vatine et al., 2013), and we have shown that zebrafish *mct8* is primarily expressed in neurons, glial cells and the vascular system, as is the case in humans (Vatine et al., 2013; Zada et al., 2014). Furthermore, *mct8* mutant (*mct8*^−/−^) zebrafish demonstrated behavioral and neurological abnormalities, including the altered expression of myelin-related genes (Zada et al., 2014). Here, using transgenic zebrafish and live imaging, we studied hypomyelination in the CNS of *mct8*^−/−^ larvae, and tested the beneficial effect of putative drugs and targeted *mct8* gene therapy in the BBB on the development of oligodendrocytes in the brain and spinal cord (SC).

## RESULTS

### Loss of Mct8 alters the expression levels of markers for OPCs and mature oligodendrocytes

**Fig. 1.**
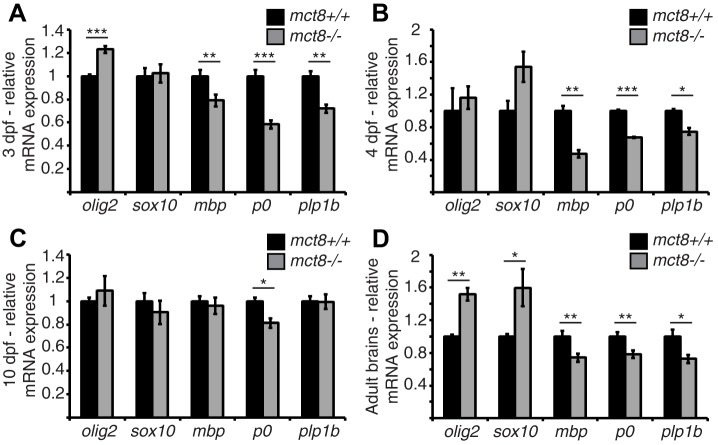
**Altered expression of myelin-related genes in *mct8***^−/−^ **zebrafish.** (A-D) Relative mRNA expression of *olig2*, *sox10*, *mbp*, *p0* and *plp1b* in 3-dpf (A), 4-dpf (B) and 10-dpf (C) larvae and in adult brains (D) of *mct8^+/+^* and *mct8*^−/−^ zebrafish. Values represent means±s.e.m. Statistical significance determined by Student's *t*-test: two samples assuming unequal variance (*P<0.05, **P<0.01, ***P<0.001).

The expression of myelin-related genes in zebrafish is first detected 2 days post-fertilization (dpf) and the onset of myelination occurs at 3 dpf, mainly in the ventral hindbrain and the SC (Brösamle and Halpern, 2002; Buckley et al., 2010a; Kirby et al., 2006). To test the effect of Mct8 elimination on myelination, the transcript levels of the OPC markers *oligodendrocyte lineage transcription factor 2* (*olig2*) and *sex determining region Y-BOX 10* (*sox10*), and the mature-oligodendrocyte markers *myelin basic protein* (*mbp*), *protein zero* (*p0*) and *proteolipid protein 1b* (*plp1b*) were quantified in 3- and 4-dpf *mct8*^−/−^ and *mct8*^+/+^ whole embryos and larvae. Whereas the mRNA levels of *sox10* did not change in *mct8*^−/−^ embryos, the mRNA levels of *olig2* increased by 22% [*t=*−6.69, degrees of freedom (d.f.)*=*14, *P<*0.001, Fig. 1A], and the mRNA levels of *mbp*, *p0* and *plp1b* were reduced by 21% (*t=*2.9, d.f.*=*18, *P<*0.01, Fig. 1A), 42% (*t=*6.45, d.f.*=*16, *P<*0.001, Fig. 1A) and 29% (*t=*5, d.f.*=*8, *P<*0.01, Fig. 1A), respectively, in *mct8*^−/−^ compared with *mct8*^+/+^ embryos. Similarly, in 4-dpf larvae, the mRNA levels of *mbp*, *p0* and *plp1b* were reduced by 53% (*t=*6.59, d.f.*=*4, *P<*0.01, Fig. 1B), 33% (*t=*19.9, d.f.*=*3, *P<*0.001, Fig. 1B) and 26% (*t=*5.18, d.f.*=*3, *P<*0.05, Fig. 1B), respectively, in *mct8*^−/−^ compared with *mct8*^+/+^ larvae. These results suggest a global increase in the number of OPCs alone, with a decrease in the number of mature oligodendrocytes in *mct8*^−/−^ embryos.

Myelin deficiencies are found in both young and mature AHDS patients (Gika et al., 2010; Holden et al., 2005; La Piana et al., 2015). Thus, we also measured the expression levels of myelin marker genes in 10-dpf larvae and adult brains. Similar to 3- and 4-dpf embryos (Fig. 1A), the mRNA levels of *olig2* and *sox10* increased by 51% (*t=*−6.4, d.f.*=*2, *P<*0.05, Fig. 1D) and 59% (*t=*−2.62, d.f.*=*5, *P<*0.05, Fig. 1D), respectively, and *mbp*, *p0* and *plp1b* mRNA levels were reduced by 26% (*t=*3.12, d.f.*=*16, *P<*0.01, Fig. 1D), 22% (*t=*3.08, d.f.*=*13, *P<*0.01, Fig. 1D) and 28% (*t=*2.74, d.f.*=*12, *P<*0.05, Fig. 1D), respectively, in *mct8*^−/−^ compared with *mct8*^+/+^ adult brains. In contrast, in 10-dpf *mct8*^−/−^ larvae, the loss of Mct8 did not affect the expression of the markers, excluding *p0*, which is expressed specifically in the CNS (Brösamle and Halpern, 2002; Zada et al., 2014) and was reduced by 19% (*t=*3.1, d.f.*=*4, *P<*0.05, Fig. 1C) in *mct8*^−/−^ compared with *mct8*^+/+^ larvae. Altogether, these results suggest reduced differentiation of OPCs into mature oligodendrocytes in the CNS.

### Visualizing myelination and glial cell dynamics in live *mct8*^−/−^ larvae

**Fig. 2.**
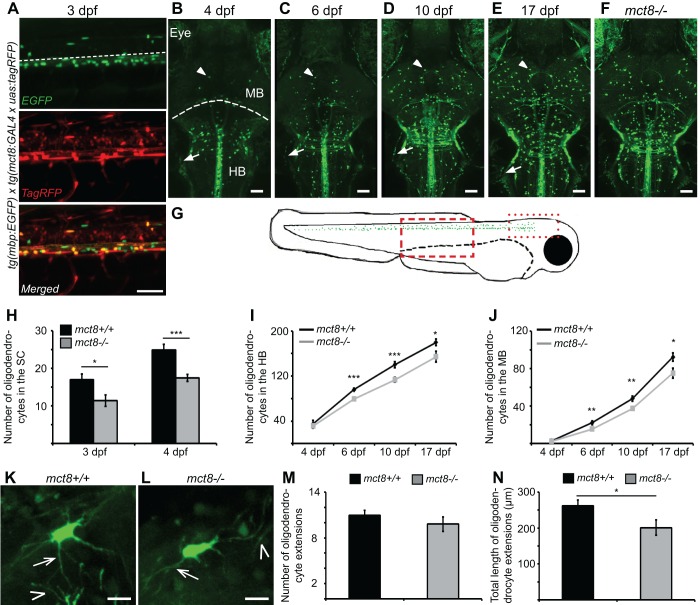
**Hypomyelination in the brain and spinal cord (SC) of *mct8^−/−^* larvae during development.** (A) Lateral view of the trunk in 3-dpf *tg(mbp:EGFP) x tg(mct8:GAL4* *x* *uas:tagRFP)* embryo revealed colocalization of *mct8*-promoter-driven signal (red, middle panel) in oligodendrocytes (green, top panel) of the SC. Dashed white line in the top picture marks the boundary between the dorsal and ventral SC. (B-F) Dorsal view of the hindbrain (HB) and midbrain (MB; dashed white line marks the boundary between the MB and HB) of 4 (B), 6 (C), 10 (D) and 17 (E)-dpf *tg(mbp:EGFP)* larvae, as well as 17-dpf *tg(mbp:EGFP)/mct8*^−/−^ larvae (F). Arrows and arrowheads indicate Schwann cells and oligodendrocytes, respectively. (G) Illustration of the *tg(mbp:EGFP)* larvae. Distribution of oligodendrocyte in the CNS is marked with green spots. Dashed and dotted boxes represent the imaged areas in the SC and brain, respectively, as shown in A-F. (H) Number of oligodendrocytes in the dorsal SC of 3 (*mct8^+/+^*: *n*=23, *mct8^−/−^*: *n*=23)- and 4 (*mct8^+/+^*: *n*=19, *mct8*^−/−^: *n*=21)-dpf larvae. (I) Number of oligodendrocytes counted in the HB of 4 (*mct8*^+/+^: *n*=7, *mct8*^−/−^: *n*=14)-, 6 (*mct8*^+/+^: *n*=33, *mct8*^−/−^: *n*=39)-, 10 (*mct8*^+/+^: *n*=17, *mct8*^−/−^: *n*=18)- and 17 (*mct8*^+/+^: *n*=14, *mct8^−/−^*: *n*=12)-dpf larvae. (J) Number of oligodendrocytes counted in the MB of 4 (*mct8*^+/+^: *n*=8, *mct8*^−/−^: *n*=8)-, 6 (*mct8*^+/+^: *n*=25, *mct8*^−/−^: *n*=30)-, 10 (*mct8*^+/+^: *n*=17, *mct8*^−/−^: *n*=18)- and 17 (*mct8*^+/+^: *n*=13, *mct8*^−/−^: *n*=13)-dpf larvae. (K,L) Imaging of single oligodendrocytes in the MB of *mct8*^+/+^ (K) and *mct8*^−/−^ (L) 10-dpf larvae. Arrows and arrowheads indicate self and neighboring oligodendrocyte extensions, respectively. (M) The number of oligodendrocyte extensions per single cell (*mct8*^+/+^: *n*=29 cells, *mct8*^−/−^: *n*=24 cells). (M) Total length of oligodendrocyte extensions per single cell (*mct8*^+/+^: *n*=29 cells, *mct8*^−/−^: *n*=24 cells). Scale bars: 50 µm (A-F), 10 µm (K,L). Values are represented as means±s.e.m. Statistical significance was determined by Student's *t*-test: two samples assuming unequal variance (*P<0.05, **P<0.01, ***P<0.001).

**Fig. 3.**
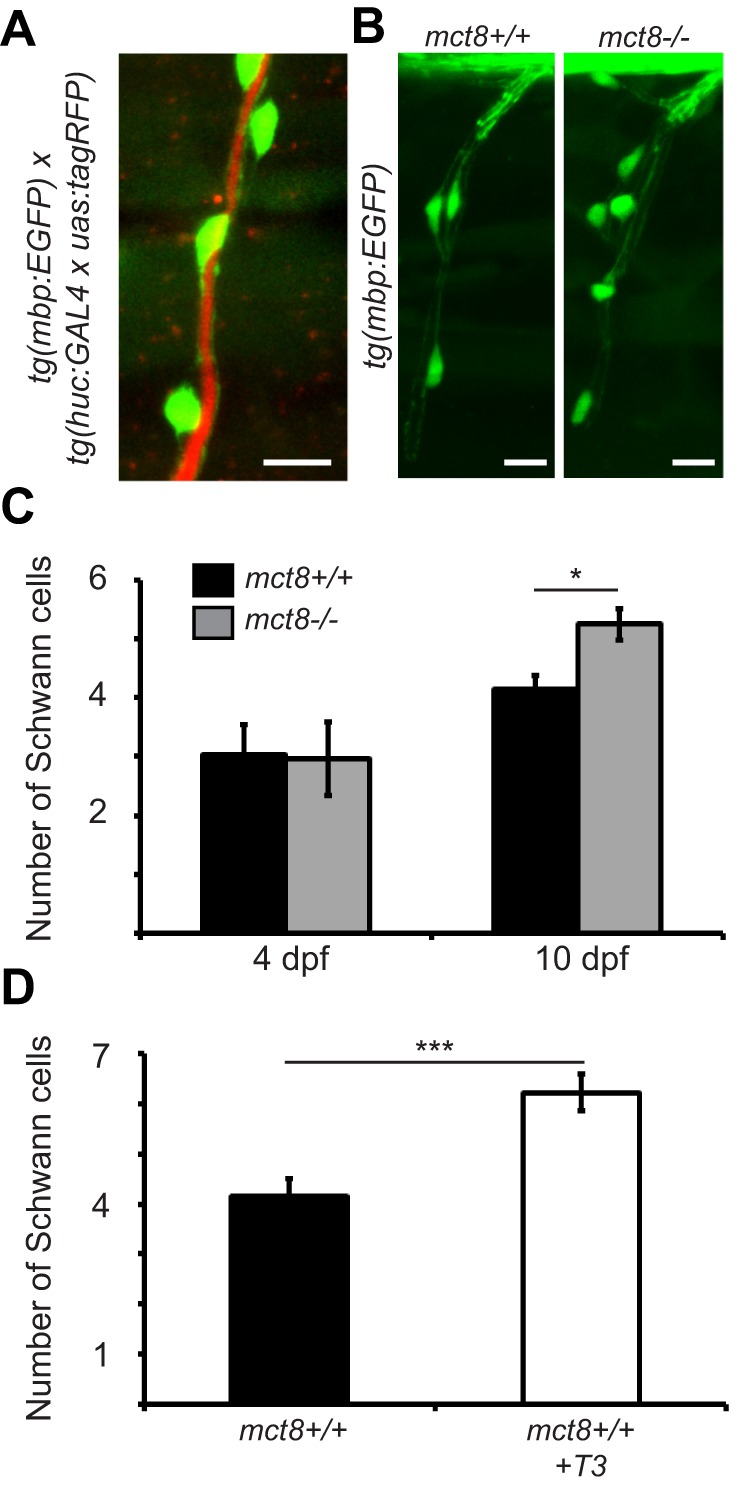
**Increased number of Schwann cells in *mct8***^−/−^ **larvae during development.** (A) Lateral view of a motor neuron in 10-dpf live *tg(mbp:EGFP)/tg(huc:GAL4 x uas:tagRFP)* larvae shows Schwann cell (green) ensheathment of motor neuron axons (red). (B) Representative lateral-view images of Schwann cell ensheathment of motor neuron axons in the SC of 10-dpf *mct8*^+/+^ and *mct8*^−/−^ larvae. (C) Number of Schwann cells that myelinate the axons of the motor neurons of 4 (*mct8*^+/+^: *n*=9, *mct8*^−/−^: *n*=9)- and 10 (*mct8*^+/+^: *n*=26, *mct8*^−/−^: *n*=22)-dpf larvae. (D) Number of Schwann cells that myelinate the axons of the motor neurons of untreated and T3-treated *mct8^+/+^* 10-dpf larvae (untreated: *n*=26, T3-treated: *n*=19). Values are represented as means±s.e.m. Statistical significance was determined by Student's *t*-test: two samples assuming unequal variance (*P<0.05, ***P<0.001). Scale bars: 10 µm.

The quantification of genetic markers for glial cells in the entire body hinted for hypomyelination in *mct8*^−/−^ zebrafish. However, live imaging of specific tissues in single-cell resolution is essential to pinpoint the spatial location of the deficiency and to visualize oligodendrocyte and Schwann cell developmental dynamics. Thus, we imaged glial cell development in 3-, 4-, 6-, 10- and 17-dpf *tg(mbp:EGFP)* zebrafish, which show EGFP expression in oligodendrocytes and Schwann cells in the CNS and PNS, respectively (Jung et al., 2010). Double-transgenic assays in 3-dpf progeny of *tg(mct8:GAL4* *x* *uas:tagRFP)* and *tg(mbp:EGFP)* zebrafish confirmed that *mct8* is expressed in some, but not all, *mbp*-positive cells (Fig. 2A). We then characterized the development of the glial cell. In the trunk, a large number of glial cells are located in the ventral part of the SC, and some oligodendrocytes migrated dorsally during development (Fig. 2A). At 4 dpf, Schwann cells migrated ventrally along the motor neuron axons, and myelin sheaths were clearly visible in 10-dpf larvae (Fig. 3A). At 3 dpf, a small number of oligodendrocytes in the brain were apparent, mainly around the midline, at the ventral hindbrain (Fig. 5D). At 4 dpf, the number of cells increased, and the cells were distributed primarily in the hindbrain and midbrain (Fig. 2B). This developmental tendency persisted, and a growing number of oligodendrocytes were distributed in the midbrain, hindbrain and SC in 6-, 10- and 17-dpf larvae (Fig. 2C-E). These results show that oligodendrocytes and Schwann cells first appear in 3-dpf and 4-dpf embryos, respectively, and robust differentiation and proliferation of glial cells, as well as myelination, occurs during development in the brain and SC.

### The number of oligodendrocytes is reduced in *tg(mbp:EGFP)/mct8*^−/−^ zebrafish

The gene expression results (Fig. 1) suggest that the hypomyelination process occurs in the CNS of *mct8*^−/−^ embryos and adults. In order to determine whether the number of oligodendrocytes is affected by loss of Mct8, we counted the number of oligodendrocytes in the brain and SC of *mct8*^−/−^ and *mct8*^+/+^ embryos and larvae. To quantify the cell number, adult *tg(mbp:EGFP)*/*mct8*^+/−^ and *mct8*^+/−^ zebrafish were crossed, and the number of cells counted (Fig. 2A-G). This approach assures that all progeny carry the same transgene, and enables comparison between sibling EGFP-positive and -negative embryos. Image analysis showed that the number of oligodendrocytes in the dorsal SC of 3- and 4-dpf *tg(mbp:EGFP)*/*mct8*^−/−^ larvae was reduced by 33% (*t*=2.6, d.f.=44, *P*<0.05, Fig. 2H) and 30% (*t*=4.35, d.f.=33, *P*<0.001, Fig. 2H), respectively. In the brain, the number of oligodendrocytes did not change in 4-dpf *tg(mbp:EGFP)/mct8*^−/−^ embryos, presumably because OPCs only start their differentiation at this relatively early developmental stage. In the hindbrain of 6- and 10-dpf *tg(mbp:EGFP)*/*mct8*^−/−^ larvae, the number of oligodendrocytes was reduced by 19% (*t*=4.39, d.f.=70, *P*<0.001, Fig. 2I) and 20% (*t*=3.86, d.f.=32, *P*<0.001, Fig. 2I), respectively. Similarly, in the midbrain, the number of cells was reduced by 30% (*t*=2.8, d.f.=45, *P*<0.01, Fig. 2J) and 23% (*t*=3.18, d.f.=29, *P*<0.01, Fig. 2J), respectively, in *tg(mbp:EGFP)*/*mct8*^−/−^ larvae compared with wild type. In older (17 dpf) *tg(mbp:EGFP)*/*mct8*^−/−^ larvae (Fig. 2E,F), the number of oligodendrocytes in the hindbrain and midbrain was reduced by 16% (*t*=2.23, d.f.=18, *P*<0.05, Fig. 2I) and 20% (*t*=2.49, d.f.=23, *P*<0.05, Fig. 2J), respectively.

The reduced number of oligodendrocytes in the CNS suggests that loss of Mct8 causes hypomyelination in *mct8*^−/−^ zebrafish. However, since a substantial amount of oligodendrocytes remained intact in *mct8*^−/−^ larvae, they could compensate for the loss of cells and generate either more or longer cell extensions that produce the myelin sheaths. To study the morphology of the oligodendrocytes, we imaged single cells in the midbrain of 10-dpf *mct8*^−/−^ and *mct8*^+/+^ larvae. Although the number of extensions in a single oligodendrocyte was similar in both genotypes (Fig. 2K-M), the total length of the extensions was reduced by 23% (*t*=2.22, d.f.=45, *P*<0.05, Fig. 2K,L,N) in *mct8*^−/−^ compared to *mct8*^+/+^ larvae. Moreover, extensions of adjacent cells were more visible next to the cell soma of *mct8*^+/+^ compared to *mct8*^−/−^ larvae (Fig. 2K,L). These results show that a loss of Mct8 results in a reduction in the number of oligodendrocytes during development and a lower density of oligodendrocyte extensions, and establishes the *tg(mbp:EGFP)*/*mct8*^−/−^ zebrafish as a model for hypomyelination.

### The number of Schwann cells increased in *tg(mbp:EGFP)/mct8*^−/−^ zebrafish

The levels of *mbp* mRNA in the whole larvae did not change (Fig. 1B), whereas the number of oligodendrocytes in the CNS decreased (Fig. 2I,J) in 10-dpf *mct8*^−/−^ larvae. Because *mbp* is a marker of myelin in the CNS and PNS, we imaged *tg(mbp:EGFP)/mct8*^−/−^ zebrafish and tested the effect of Mct8 elimination on the development of Schwann cells in the periphery (Fig. 3A). Whereas the number of Schwann cells did not change in 4-dpf *tg(mbp:EGFP)*/*mct8*^−/−^ larvae, their number increased by 26% (*t*=−3.17, d.f.=43, *P*<0.05, Fig. 3B,C) in 10-dpf *tg(mbp:EGFP)/mct8*^−/−^ larvae. These results suggest that Mct8 elimination causes a hyperthyroid state in the periphery that in turn induces Schwann cell maturation. Thus, we tested the effect of T3 treatment on the number of Schwann cells in 10-dpf *mct8*^+/+^ larvae. Following treatment for 4 consecutive days, the number of Schwann cells increased by 49% (*t*=−4.84, d.f.=31, *P*<0.001, Fig. 3D) compared to untreated *mct8*^+/+^ larvae. Taken together, these results show a decrease in oligodendrocyte number in the CNS and an increase in Schwann cell number in the PNS in *mct8*^−/−^ larvae. These myelin deficiencies correlate with the brain hypothyroidism and periphery hyperthyroidism found in AHDS (Ferrara et al., 2013; Trajkovic et al., 2007). Thus, the *tg(mbp:EGFP)/mct8*^−/−^ zebrafish can be used to assay the effect of TH-related pharmacological and genetic treatments on altered myelination in AHDS.

### TH analogs and clemastine rescue hypomyelination in *mct8*^−/−^ zebrafish

Establishing the myelination deficiencies in *tg(mbp:EGFP)/mct8*^−/−^ larvae, we tested putative drugs that might rescue this phenotype. *In vitro* and *in vivo* studies in mammals have demonstrated that TH analogs, i.e. 3,3′,5-triiodothyroacetic acid (TRIAC; TA3) and 3,5-diiodothyropropionic acid (DITPA), can cross the cell membrane even in the absence of Mct8 (Di Cosmo et al., 2009; Kersseboom et al., 2014; Messier and Langlois, 2000; Verge et al., 2012; Verhoeven et al., 2002). In addition, clemastine (also named Tavist), which is an antihistamine drug, can enhance oligodendrocyte differentiation and the wrapping of micropillars in cell lines and mice (Mei et al., 2014). Thus, we tested the potential beneficial effect of these drugs on CNS hypomyelination in the *tg(mbp:EGFP)/mct8*^−/−^ larvae.

**Fig. 4.**
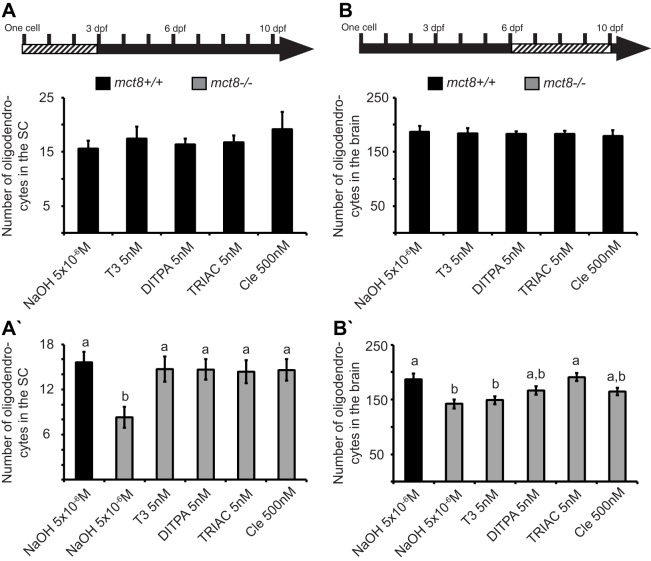
**TH-analog and clemastine treatments rescue the number of oligodendrocytes in *mct8***^−/−^ **larvae.** The arrows at the top of the figure represent the developmental timeline of the experiment. Dashed bar represents the period in which the drug was administered. (A,A′) Number of oligodendrocytes in the spinal cord (SC) of 3-dpf *mct8*^+/+^ embryos treated with T3 (*n*=11), DITPA (*n*=12), TRIAC (*n*=11), clemastine (Cle, *n*=10) or NaOH (*n*=16), and *mct8*^−/−^ embryos treated with T3 (*n*=12), DITPA (*n*=14), TRIAC (*n*=14), clemastine (Cle, *n*=12) or NaOH (*n*=16) (*P*<0.01 for ‘a’ and ‘b’). (B,B′) Number of oligodendrocytes in the brain of 10-dpf *mct8*^+/+^ larvae treated with T3 (*n*=10), DITPA (*n*=11), TRIAC (*n*=11), clemastine (Cle, *n*=10) or NaOH (*n*=10), and *mct8*^−/−^ larvae treated with T3 (*n*=10), DITPA (*n*=12), TRIAC (*n*=11), clemastine (Cle, *n*=15) or NaOH (*n*=13, *P*<0.001 for ‘a’ and ‘b’). Values represented as means±s.e.m. Statistical significance determined by one-way ANOVA, followed by post-hoc Tukey HSD test. The letters above the bars indicate significant differences.

We exposed *tg(mbp:EGFP)/mct8*^−/−^ and *tg(mbp:EGFP)/mct8*^+/+^ embryos and larvae to 5×10^−6^ M control NaOH, 5 nM T3, DITPA or TRIAC, or 500 nM clemastine. These compound concentrations were chosen based on pre-calibration assays, where the highest doses that did not affect pigmentation and the general morphology of the embryos were selected. Initially, the drugs were administered into the water at the one-cell stage, and the treatment lasted 3 consecutive days. At 3 dpf, the number of oligodendrocytes in the dorsal SC did not change in all treated *tg(mbp:EGFP)*/*mct8^+/+^* embryos compared with control *tg(mbp:EGFP)*/*mct8*^+/+^ embryos (Fig. 4A). However, whereas the oligodendrocyte number was reduced by 47% in control *tg(mbp:EGFP)/mct8*^−/−^ 3-dpf embryos (*F*=3.78, d.f.=5, *P*<0.01, Fig. 4A′), their number increased and they were completely rescued in T3-, DITPA-, TRIAC- and clemastine-treated *tg(mbp:EGFP)/mct8*^−/−^ embryos compared with the control *tg(mbp:EGFP)/mct8*^+/+^ embryos (*P*<0.01, Fig. 4A′). These results suggest that treatment immediately following fertilization can prevent the myelination deficiencies in *mct8*^−/−^ larvae.

In order to determine whether these drugs can repair, and not only prevent, the myelination deficiencies at the developmental stage, when the oligodendrocyte number is already reduced, we exposed 6-dpf larvae to these compounds for 4 consecutive days. At 10 dpf, the number of oligodendrocytes in the brain did not change in all treated *tg(mbp:EGFP)*/*mct8*^+/+^ larvae compared with control *tg(mbp:EGFP)*/*mct8*^+/+^ larvae (Fig. 4B). However, whereas the oligodendrocyte number was reduced by 25% in control *tg(mbp:EGFP)*/*mct8*^−/−^ larvae (*F*=5.75, d.f.=5, *P*<0.001, Fig. 4B′), their number increased and was completely rescued in TRIAC-treated *tg(mbp:EGFP)*/*mct8*^−/−^ larvae compared with the control *tg(mbp:EGFP)*/*mct8*^−/−^ larvae (*P*<0.001, Fig. 4B′). Furthermore, DITPA and clemastine partially rescued oligodendrocyte number in *tg(mbp:EGFP)*/*mct8*^−/−^ larvae (Fig. 4B′). In contrast, oligodendrocyte number did not change in T3-treated *mct8*^−/−^ larvae. These results show that the TH analogs and clemastine can enhance myelination in *tg(mbp:EGFP)*/*mct8*^−/−^ larvae even after brain damage occurs. In addition, these results suggest that T3 cannot cross the cell membrane and enter into the CNS following the 6-dpf developmental stage, and, therefore, cannot rescue myelination deficiencies in 10-dpf *mct8*^−/−^ larvae.

### Targeted gene therapy to the BBB rescues hypomyelination in *mct8*^−/−^ zebrafish

The BBB is vital for the maintenance and protection of the brain. This barrier is formed by a tight junction between endothelial cells of the vascular system. In zebrafish, the BBB is first detected in 3-dpf embryos, and its maturation occurs between 3 and 10 dpf (Fleming et al., 2013; Jeong et al., 2008). Using the endothelial-cell-specific *fli* promoter (Lawson and Weinstein, 2002; Yaniv et al., 2006) and the double-transgenic *tg(mct8:EGFP/fli:dsRED)* larvae, we showed that Mct8 is widely expressed in the vascular system of the larval trunk and brain (Vatine et al., 2013). Considering the efficiency and ineffectiveness of T3 treatment in 3- and 10-dpf larvae, respectively (Fig. 4A′,B′), as well as the maturation of the BBB between these developmental stages, we reasoned that Mct8 expression in endothelial cells of the BBB is crucial for T3 transport and normal brain development. Thus, specific expression of *mct8* in endothelial cells of *mct8*^−/−^ larvae might enable endogenous T3 to cross the BBB, enter into the hypothyroidized brain and rescue myelination deficiencies.

**Fig. 5.**
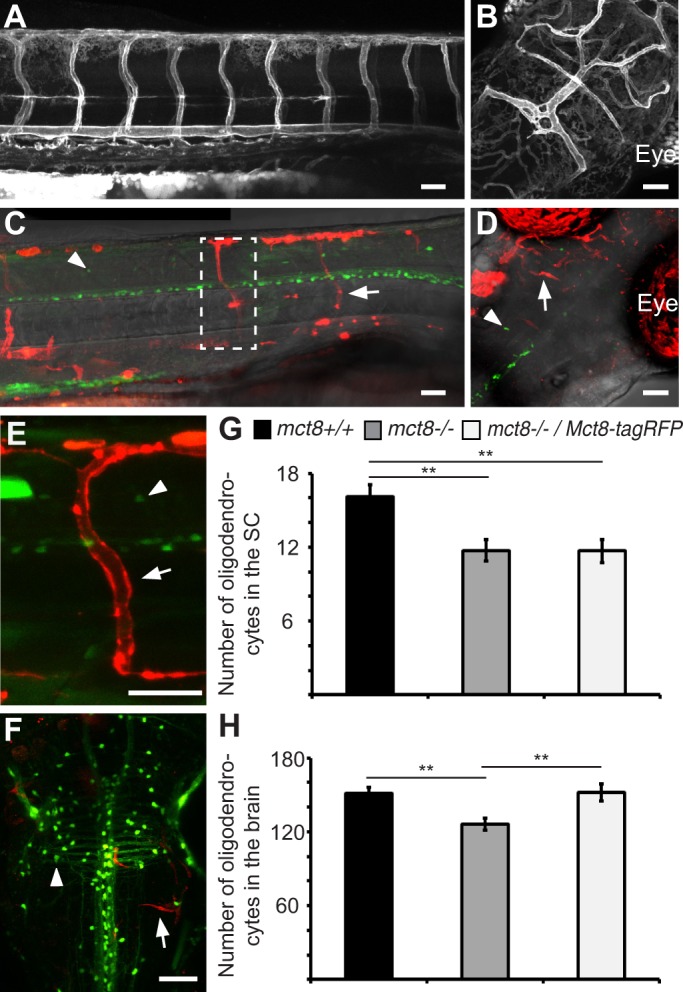
**Specific expression of Mct8 in the vascular system and BBB rescues the number of oligodendrocytes in the brain.** (A,B) Lateral view of the trunk (A, head pointing to the right) and dorsal view of the head (B) of a *tg(fli:Mct8-EGFP)* 3-dpf embryo. (C-F) Lateral view of the trunk (C,E, head pointing to the right) and dorsal view of the head (D,F) of a *tg(mbp:EGFP)* larvae injected with *fli:Mct8-tagRFP*. Arrows indicate the expression of Mct8-tagRFP in the endothelial cells of the vascular system, and arrowheads indicate oligodendrocytes in the CNS. Wide (C) and high-magnification (dashed box in C, E) views in 3-dpf larvae show red mosaic expression of Mct8-tagRFP and green oligodendrocytes. Dorsal view of the head shows green oligodendrocytes in the brain and mosaic expression of Mct8-tagRFP in 3 (D)- and 10 (F)-dpf larvae. (G) Number of oligodendrocytes counted in the spinal cord (SC) of 3-dpf larvae (*mct8*^+/+^: *n*=37, *mct8*^−/−^: *n*=42 and *mct8*^−/−^*/Mct8-tagRFP*: *n*=26, **P<0.01). (H) Number of oligodendrocytes counted in the brain of 10-dpf larvae (*mct8*^+/+^: *n*=10, *mct8*^−/−^: *n*=10 and *mct8*^−/−^*/Mct8-tagRFP*: *n*=11, **P<0.01). Values represented as means±s.e.m. Statistical significance determined by one-way ANOVA followed by post-hoc Tukey HSD test (**P<0.01). Scale bars: 50 µm.

In order to test this assumption, the *pT2-fli:Mct8-tagRFP* construct, which drives specific expression of the fusion protein Mct8-tagRFP in vascular endothelial cells, was injected into one-cell-stage *tg(mbp:EGFP)/mct8*^−/−^ and *tg(mbp:EGFP)/mct8*^+/+^ embryos, and 3-dpf Mct8-tagRFP-positive embryos were imaged and sorted. In the head and trunk, the mosaic expression of Mct8-tagRFP was apparent in approximately 10-20% of the vascular system (Fig. 5C-F), compared to stable expression of Mct8-EGFP in the entire vascular system of 3-dpf *tg(fli:Mct8-EGFP)* embryos (Fig. 5A,B). At 3 dpf, as expected, the number of oligodendrocytes was reduced by 28% (*F*=6.99, d.f.=2, *P*<0.01, Fig. 5G) in the spinal cord of *tg(mbp:EGFP)/mct8*^−/−^ compared with *tg(mbp:EGFP)/mct8*^+/+^ larvae. Notably, the number of oligodendrocytes in the SC of *tg(mbp:EGFP)/mct8*^−/−^*/fli:Mct8-tagRFP-*positive embryos did not change compared to *tg(mbp:EGFP)/mct8*^−/−^ embryos (Fig. 5G). These results show that the expression of Mct8 in the vascular endothelial cells does not affect myelination in 3-dpf embryos. Following the imaging at 3 dpf, the same individual embryos were raised and subjected to live imaging at 10 dpf. Remarkably, whereas the number of oligodendrocytes in the brain of *tg(mbp:EGFP)/mct8*^−/−^ larvae was reduced by 19% (*F*=8.74, d.f.=2, *P*<0.01, Fig. 5F,H), the number of oligodendrocytes in *tg(mbp:EGFP)/mct8*^−/−^*/fli:Mct8-tagRFP-*positive larvae was completely recovered, and was similar to the number of oligodendrocytes in *tg(mbp:EGFP)/mct8*^+/+^ larvae (*P*<0.01, Fig. 5F,H). These results show that Mct8 expression in the vascular endothelial cells can rescue hypomyelination in 10-dpf *mct8*^−/−^ larvae. Considering that specific Mct8 expression in endothelial cells did not rescue myelination in 3-dpf larvae before the development of the BBB, these results demonstrate that this genetic treatment is beneficial post-damage and that Mct8 function specifically in the BBB is crucial for the brain. Altogether, the results in the *mct8*^−/−^ zebrafish model suggest that BBB-specific *mct8* gene therapy is a promising potential treatment for AHDS patients.

## DISCUSSION

Understanding the cellular mechanism underlying leukodystrophies and establishing new animal models for these disorders are expected to enhance the discovery of novel treatments. In this study, we characterized hypomyelination in a zebrafish model for AHDS and used the transgenesis and imaging of live larvae in order to evaluate the therapeutic effect of various genetic and pharmacological treatments. Quantification of the expression of myelin-related markers in *mct8*^−/−^ larvae implied at the delayed maturation of oligodendrocytes and altered myelination. Indeed, the number of oligodendrocytes in the brain and spinal cord was reduced, whereas the number of Schwann cells in the trunk increased in *mct8*^−/−^ larvae. In order to test potential treatments, pharmacological and genetic approaches were both evaluated. The hypomyelination was partially or completely repaired by TH analogs and clemastine, even when the drugs were administered after the deficiency was already apparent. In addition, Mct8 expression that was specifically targeted to the endothelial cells of the vascular system completely restored myelination in *mct8^−/−^* larvae only at the developmental stage, when the BBB was already established. Thus, we propose pharmacological treatment and BBB-targeted gene therapy as potential therapeutics for hypomyelination in AHDS patients.

Myelination primarily occurs in mammals during postnatal stages, whereas, in zebrafish, the first OPCs emerge at 2 dpf and oligodendrocytes appear shortly after (Brösamle and Halpern, 2002; Buckley et al., 2010a; Park et al., 2002). The myelination process continues during development even in adults in all vertebrates, including zebrafish, rodents and humans (Jung et al., 2010; Miller et al., 2012). However, the techniques by which myelination is quantified vary between species and studies. One of the most adequate anatomical methods is electron microscopy (EM); however, EM imaging is performed in fixed brain sections, whereas neurons and axons stand out in their significant capacity to continuously change, adapt and develop. Thus, using EM to image an entire myelinated neuronal circuit across several time points and in several individuals on a large scale is virtually impossible (Buckley et al., 2010a); therefore, an alternative framework for studying myelination that will include time-lapse live imaging could overcome these limitations. Here, we combined the expression assays of myelin markers with the visualization of single oligodendrocytes or Schwann cells in live transgenic zebrafish. These had already been proved to be effective and rapid methods to examine myelination in many animals during development (Pogoda et al., 2006; Langworthy and Appel, 2012; Buckley et al., 2010b). Using these methods, we found a gradual increase in the number of mature oligodendrocytes that initially appear in the ventral hindbrain and spinal cord of 3-dpf embryos and extend their distribution into other brain regions at early and late larval stages. This pattern of development, as well as the unique genetic and imaging tools used in the zebrafish model, provided us with a suitable platform to understand the cellular mechanism of hypomyelination in AHDS.

Brain MRI scans of AHDS patients show profound myelination deficiencies in the central and peripheral white matter of the frontal, parietal and temporal lobes (Gika et al., 2010). Moreover, *Mct8/Oatp1c1* dKO mice display a reduced MBP immunofluorescence signal in the cerebral cortex and a reduced number of myelinated axons in the corpus callosom (Mayerl et al., 2014). Surprisingly, although hypomyelination is evident, the ultrastructure of the myelin sheath seems to be normal in this area. Similar to the finding in AHDS patients and *Mct8/Oatp1c1* dKO mice, we found decreased expression of markers for myelin and a reduced number of oligodendrocytes in the brain and spinal cord. Thus, these results suggest that hypomyelination in animal models and individuals with AHDS is caused by the inhibition of OPC differentiation and, ultimately, a reduced number of oligodendrocytes, while, in regions where the amount of oligodendrocytes is intact, the structure and function of the myelin sheath is normal. Thus, unlike neurodegenerative diseases in which cells are eliminated, in AHDS, the promotion of OPC maturation and oligodendrocyte migration toward deficient regions is a promising therapeutic approach.

An intriguing finding is the difference between the decrease in the number of oligodendrocytes found in the CNS and the increase in the number of Schwan cells found in the periphery in *mct8*^−/−^ larvae. Furthermore, whereas the expression of markers for myelin decreases in adult brains and 3-dpf *mct8*^−/−^ embryos (at a stage at which myelin appears only in the brain), it did not change in 10-dpf larvae (at a stage at which myelin is distributed throughout the CNS and PNS). This can be explained by the deferential TH levels found in MCT8-deficient animals – hypothyroidism in the CNS and hyperthyroidism in the periphery (Ferrara et al., 2013; Trajkovic et al., 2007), which correlate with a decreased number of oligodendrocytes and an increased number of Schwann cells, respectively. Indeed, we found that T3 promotes maturation of Schwann cells in larvae. Supporting this notion, TH regulates the timing of oligodendrocyte differentiation (Bernal, 2000; Billon et al., 2001) and promotes myelination by inducing the expression of genes such as *mbp* and *p0* (Barradas et al., 2001; Zada et al., 2014)*.* Accordingly, normalizing TH levels in a specific tissue could repair the myelin deficiencies in MCT8-deficient animals.

Why does the elimination of MCT8 cause hypomyelination? A reasonable explanation is that there is a lack of the myelin-promoting TH in the CNS. Similar to the function of transcription factors, TH binds to nuclear TH receptor, which in turn binds the regulatory region of myelin-related genes and enhances transcriptions (Zada et al., 2014; Knipper et al., 1998; Jeannin et al., 1998; Harsan et al., 2008). Thus, in the absence of Mct8, the expression levels of these genes are altered, resulting in the abnormal development of myelin. Supporting this explanation, in individuals with AHDS, it is assumed that lack of T3 entrance into the CNS results in a global lack of white matter (Gika et al., 2010; Holden et al., 2005; La Piana et al., 2015). Furthermore, loss of MCT8 results in hypothyroid and hyperthyroid states in the CNS and the peripheral tissues of rodents (Trajkovic et al., 2007), and TH promotes the differentiation of OPCs (Bernal, 2000). Furthermore, we showed that the treatment of *mct8*^−/−^ zebrafish with TH analogs, which can enter the cell in the absence of Mct8, partially or completely rescued hypomyelination. Thus, a loss of Mct8 likely affects myelination by TH signaling; however, an alternative role for Mct8 cannot be ruled out.

The pharmacological studies on zebrafish not only enhance understanding of the mechanism underlying hypomyelination in AHDS, but also provide robust quantitative assays to evaluate the efficiency of many putative drugs. In a previous study, we showed that the TH analogs DITPA and TRIAC recovered the expression of the myelin marker *p0* in 3-dpf *mct8*^−/−^ embryos (Zada et al., 2014). Here, in addition to the quantification of genetic myelin-cell markers, we established a live-imaging-based assay that enables comparison of the effect of dozens of putative drugs on myelination. A prominent advantage of the zebrafish model is the ability to apply each drug directly to the water in a 96-well plate filled with embryos at various developmental stages and genetic backgrounds. Taking advantage of this robust assay in live animals, we tested the effect of the TH analogs DITPA and TRIAC as potential treatments for hypomyelination in AHDS, and found that both drugs can prevent the occurrence of hypomyelination in *mct8^−/−^* embryos, and TRIAC can completely rescue hypomyelination in the larvae even after damage was apparent. This suggests TRIAC as a promising treatment for TH-dependent hypomyelination. However, considering that DITPA partially rescued hypomyelination, further assays with various concentrations and at various developmental stages are required. Supporting the results in zebrafish, the administration of TRIAC for 12 consecutive postnatal days was found to improve cerebellar development and cortical myelination in *Mct8/Oatp1c1* dKO mice (Kersseboom et al., 2014; Visser et al., 2016). DITPA, on the other hand, improves hypermetabolism and TH-level abnormalities in the serum and brain of *Mct8*-KO mice (Ferrara et al., 2014, 2015; Verge et al., 2012).

In addition to TH analogs, the *tg(mbp:EGFP)/mct8*^−/−^ model was used to show the beneficial effect of clemastine on hypomyelination. To date, this myelin-promoting drug was tested only *in vitro* and in mice with toxic injury in the spinal cord white matter tracts (Mei et al., 2014). Clearly, further clemastine studies, including treatment with various concentrations at several developmental stages, are needed. Nevertheless, these findings show that clemastine enhances the differentiation of oligodendrocytes in a zebrafish model for AHDS, and suggest that clemastine, as well as TH analog treatments, can enhance myelination in other leukodystrophies.

In vertebrates, MCT8 is expressed in neurons, glial cells, and endothelial cells of the vascular system, including in the BBB (Friesema et al., 2012; Müller and Heuer, 2014; Pizzagalli et al., 2002; Roberts et al., 2008; Vatine et al., 2013). In contrast to humans, the TH transporter OATP1C1 is profoundly expressed in the BBB of mice. Interestingly, KO of both *Mct8* and *Oatp1c1* are required to mimic the brain hypothyroidism and neurological phenotype of individuals with AHDS (Mayerl et al., 2014). Thus, it was proposed that loss of MCT8 expression in the BBB is a key factor that mediates the pathology of the disease (Bernal, 2000; Mayerl et al., 2014; Visser and Visser, 2012). Supporting this hypothesis, we showed that T3 administration prevents hypomyelination in 3-dpf *mct8*^−/−^ embryos but not in 10-dpf *mct8*^−/−^ larvae, which were treated between 6 and 10 dpf. Taking into account that the maturation of the BBB in zebrafish occurs between 3 and 10 dpf (Fleming et al., 2013; Jeong et al., 2008), these results suggest that the lack of TH transport, specifically in the BBB, is a fundamental impairment in AHDS.

The idea that Mct8 expression in the BBB is key for brain function raised the possibility that restricted expression of Mct8 in the BBB of *mct8*^−/−^ larvae will benefit the brain and rescue hypomyelination. Thus, we transiently expressed Mct8 in the endothelial cells of the vascular system. Strikingly, the mosaic expression of Mct8 rescued the number of oligodendrocytes in the brain of 10-dpf *tg(mbp:EGFP)/mct8*^−/−^ larvae, but not in 3-dpf *tg(mbp:EGFP)/mct8*^−/−^ larvae. This is probably because the BBB forms and develops between 3 and 10 dpf in zebrafish. These results suggest that, although Mct8 is also expressed in neurons and glial cells, endothelial-cell-specific expression of Mct8 makes the BBB permeable to TH and enables TH access into the brain, which is sufficient to induce myelination. Recently, intravenous injection of adeno-associated virus 9 driving the expression of human MCT8 was shown to increase T3 content in the mouse brain (Iwayama et al., 2016). Further studies that test the effect of vascular Mct8 expression on neurons and glial cells in various brain regions in *mct8*^−/−^ larvae and mouse models are required in order to evaluate this treatment approach. Nevertheless, these results suggest that selective BBB transport will enable TH to reach the brain and might limit the progression of the disorder or even improve the symptoms. Thus, developing BBB-targeted Mct8 gene therapy or effective BBB TH-delivery technology is expected to be an attractive future direction in AHDS treatment.

This work established the *mct8*^−/−^ zebrafish as a model for studying the mechanism and treatment of hypomyelination in AHDS and possibly other leukodystrophies. Imaging of single glial cells in live zebrafish, as well as genetic and pharmacological manipulation, showed that TH analogs and clemastine, as well as gene therapy in the BBB, rescue myelination deficiencies in *mct8*^−/−^ larvae. Future studies that will investigate the correlation between hypomyelination and neuronal activity in various brain regions are necessary to identify deficient brain regions and to understand the link between neurological and behavioral impairments. Studies on the *mct8*^−/−^ zebrafish are expected to shed light on these brain processes because two-photon imaging of genetically encoded calcium indicators (GCaMPs), combined with silencing and activating neuronal circuits by optogenetic tools, are widely used technologies in biomedical zebrafish research (Douglass et al., 2008; Leung et al., 2013; Muto et al., 2013; Wyart and Del Bene, 2011). In addition, because zebrafish have become an ideal platform for high-throughput screens of small molecules that affect neuropsychiatric disorders (Bruni et al., 2016; Rennekamp et al., 2016), transgenic zebrafish that express glial cell markers on the genetic background of *mct8*^−/−^ zebrafish might reveal new compounds that promote OPC differentiation, oligodendrocyte maintenance and myelin recovery in AHDS and other hypomyelination disorders.

## MATERIALS AND METHODS

### Zebrafish husbandry and transgenic lines

Adult zebrafish were raised and maintained in fully automated zebrafish housing systems (Aquazone, Israel; temperature 28±0.5°C, pH 7.0, conductivity 300 μS) under 14 h light/10 h dark cycles, and fed twice a day. Embryos were produced by natural spawning and cultivated in egg water containing methylene blue (0.3 ppm) in a light-controlled incubator at 28±0.5°C, as previously described (Elbaz et al., 2012). To generate a *tg(mbp:EGFP)* transgenic line that carries the *mct8* mutation, *tg(mbp:EGFP)* zebrafish (kindly provided by Dr Cheol-Hee Kim, Chungnam National University Daejeon, Korea) were crossed with *mct8*^−/−^ zebrafish. Heterozygous *tg(mbp:EGFP)/mct8*^+/−^ zebrafish were intercrossed and produced the *tg(mbp:EGFP)/mct8*^+/+^, *tg(mbp:EGFP)/mct8*^+/−^ and *tg(mbp:EGFP)/mct8*^−/−^ lines. In live-imaging experiments, *tg(mbp:EGFP)/mct8*^+/−^ was crossed with *mct8*^+/−^, and the progeny were imaged in various developmental stages and genotyped after each experiment. In quantitative real-time PCR (qRT-PCR) and pharmacological assays, the *tg(mbp:EGFP)/mct8*^+/+^ and *tg(mbp:EGFP)/mct8*^−/−^ transgenic lines were used. To generate double-transgenic lines, the *tg(mbp:EGFP)* zebrafish was crossed with *tg(mct8:GAL4* *x* *uas:TagRFP)* or *tg(huc:GAL4* *x* *uas:TagRFP)* zebrafish (Zada et al., 2014) and their live progeny were imaged. Establishment of the *tg(fli:Mct8-EGFP)* stable transgenic line was conducted using the Tol2 system (Kawakami et al., 2004) and the *pT2-fli:Mct8-EGFP* vector. Animal protocol was reviewed and approved by the Bar-Ilan University Bioethics Committee, Protocol number 41-11-2013 (‘AHDS syndrome: mechanisms of disease and therapeutic approaches in model organisms’).

### qRT-PCR assays

Relative mRNA expression levels of *olig2*, *sox10*, *mbp*, *p0* and *plp1b* were determined using qRT-PCR. Total RNA was extracted from 3-, 4- and 10-dpf larvae and adult brains using the Direct-zol RNA MiniPrep kit (Zymo Research Corporation, Irvine, CA) according to the manufacturer's instructions. For each tested gene, a total of three to ten samples were used, and each sample contained a pool of 10-25 larvae or two or three brains. One µg mRNA was reverse-transcribed using qScript cDNA SuperMix (Quanta BioSciences, Gaithersburg, MD), and relative transcript levels were determined using the 7900HT Fast Real-Time PCR System (Applied Biosystems, Foster City, CA). Duplicates of each cDNA sample were PCR-amplified using the PerfeCTa SYBR Green FastMix (Quanta BioSciences, Gaithersburg, MD) and the following specific primers: *olig2*: 5′-CGAGTGAACTGGAATAGCCTTAC-3′ and 5′-GCTCGTGTCAGAGTCCATG-3′; *sox10*: 5′-TCAATATCCGCACCTGCAC-3′ and 5′-CGCTTATCCGTCTCGTTCAG-3′; *mbp*: 5′-GAGGAGACAAGAAGAGAAAGGG-3′ and 5′-GAAATGCACGACAGGGTTG-3′; *p0*: 5′-ACCTGTGATGCCAAGAACC-3′ and 5′-TTGCCACAACGAGGATCA-3′; *plp1b*: 5′-ACACTGTTAACGTCCTGTCAG-3′ and 5′-CTGGTGCTTTGCATATGTTGG-3′; and *β-actin*: 5′-TGAATCCCAAAGCCAACAGAG-3′ and 5′-CCAGAGTCCATCACAATACCAG-3′. The relative quantification of each gene expression was normalized against *β-actin* mRNA expression levels and subjected to the ΔΔC_T_ method.

### Pharmacological assays

In all assays, embryos were placed in glass Petri dishes (20-30 embryos per dish) containing either a specific drug or 5×10^−6^ nM NaOH as a control. The medium was changed once a day in all dishes. In clemastine experiments, stock solution of 1 mM clemastine (cat. # S1847, Sellek Chemicals, Houston, TX) was prepared and diluted in zebrafish water to the final administered concentrations. A preliminary dose-dependent (10 nM to 1 µM) assay was performed on wild-type embryos. The highest substance concentration (500 nM) that did not substantially affect the morphology and behavior of the larvae was selected. In TH-analog experiments, 5 nM T3 (cat. # T2877, Sigma-Aldrich, St Louis, MO), TRIAC (cat. # T7650, Sigma-Aldrich, St Louis, MO) and DITPA (cat. # SC-256,593, Santa Cruz Biotechnology, Dallas, TX) were used, as previously described (Zada et al., 2014).

### Mosaic expression of Mct8 in the BBB

The *fli* promoter was PCR-amplified from the *p5E-fliEP* vector (kindly provided by Dr Karina Yaniv, Weizmann Institute of Science, Israel) using the 5′-AACAAGCTTCCTTGGAGATCTCATCTTTGACCC-3′ and 5′-AATGGATCCCGCGTCTGAATTAATTCCAGCCC-3′ primers, and cloned into the *pT2-uas:Mct8-tagRFP* or *pT2-uas:Mct8-EGFP* vectors using Hin*dIII* and Bam*HI*, replacing the *uas* enhancer*.* Fifty ng/µl *pT2-fli:Mct8-tagRFP* and 50 ng/µl transposase (*TP*) mRNA were co-injected into one-cell-stage *tg(mbp:EGFP)/mct8^−/−^* or *tg(mbp:EGFP)/mct8^+/+^* embryos. At 3 dpf, the embryos were imaged under an M165FC epifluorescence stereomicroscope (Leica, Wetzlar, Germany), and embryos that showed strong Mct8-tagRFP mosaic expression in the head vascular system were sorted out and later compared to their Mct8-tagRFP-negative 3- and 10-dpf sibling larvae.

### Imaging and image analysis

To perform live-imaging experiments, larvae were anesthetized with Tricaine (0.01%) and placed in low-melting-point agarose (1.0-2.0%) in a 60-mm Petri dish filled with zebrafish water. Confocal imaging was performed using a Zeiss LSM710 upright confocal microscope (Zeiss, Oberkochen, Germany). All images were processed using ImageJ (National Institutes of Health, Bethesda, MD). Calculation of the number of *mbp-*positive cells, oligodendrocyte extensions and the length of the extensions was performed using Cell Counter and NeuronJ plugins in ImageJ software (National Institutes of Health, Bethesda, MD). We imaged a 283.4×283.4 µm area in the spinal cord and a 425.1×425.1 µm area in the hindbrain and midbrain (dashed and dotted boxes in Fig. 2G, respectively). *mbp-*positive cells (oligodendrocytes) were counted in the dorsal spinal cord of 3- and 4-dpf larvae, and in the hindbrain and midbrain of 4-, 6-, 10- and 17-dpf larvae. In the late larval stages (10 and 17 dpf), we sorted out and imaged larvae of a similar size (∼4.5 mm and ∼6.2 mm, respectively).
